# Hematological and Hematopoietic Analysis in Fish Toxicology—A Review

**DOI:** 10.3390/ani13162625

**Published:** 2023-08-14

**Authors:** Małgorzata Witeska, Elżbieta Kondera, Bartosz Bojarski

**Affiliations:** 1 Department of Ichthyology and Biotechnology in Aquaculture, Institute of Animal Science, Warsaw University of Life Sciences SGGW, 02-786 Warsaw, Poland; malgorzata_witeska@sggw.edu.pl; 2 Institute of Biological Sciences, Faculty of Exact and Natural Sciences, Siedlce University of Natural Sciences and Humanities, Prusa 14, 08-110 Siedlce, Poland; elzbieta.kondera@uph.edu.pl; 3 Department of Biodiversity Protection, Institute of Animal Reproduction and Food Research, Polish Academy of Sciences, Tuwima 10, 10-748 Olsztyn, Poland

**Keywords:** fish, blood parameters, head kidney, toxicity

## Abstract

**Simple Summary:**

Hematological analysis is one of the essential tools to assess the influence of organic and inorganic substances on fish. Thus, it is widely used in ecotoxicological and pharmacotoxicological studies. In this review, we demonstrate and discuss the use, strengths, and limitations of hematological as well as hematopoietic analysis in the context of fish toxicology. Since hematopoietic analysis can help to explain and understand the mechanisms of studied hematological changes, we highly recommend this method as a good complement to standard hematological tests.

**Abstract:**

Hematological analysis is commonly used to assess the physiological state of fish. It includes red blood cell parameters, white blood cell parameters, and the number of thrombocytes per blood volume unit. Hematological analysis is one of the basic tools (often accompanied by biochemical and histopathological analyses) to assess the influence of organic and inorganic substances on fish. It is, therefore, applicable in both ecotoxicology and pharmacotoxicology. The advantages of this research method are the lack of need for specialized laboratory equipment and low costs, and the limitations are the need for extensive experience among the personnel performing the tests. One of the recommended methods of supplementing routinely determined hematological parameters is assessing the cellular composition and activity of hematopoietic tissue. As there is very little scientific data available on the issue of the effects of xenobiotics on the cellular structure of fish head kidney hematopoietic tissue, filling this gap should be considered an urgent need. Therefore, we recommend conducting research with the simultaneous use of hematological and hematopoietic analysis as reliable and complementary methods of assessing the impact of toxic substances on fish.

## 1. Hematological Analysis in Fish Toxicology

Hematological analysis of peripheral blood parameters and quantitative evaluation of blood cell morphology are useful and relatively inexpensive tools often used in fish toxicology. Blood indices are sensitive and fast reacting biomarkers of various environmental impacts, including water pollution with toxic agents. Blood parameters reflect a wide range of physiological alterations, both adaptive and disruptive. They provide extensive information about various physiological functions as reliable biomarkers of an organism’s performance. Blood sampling is less invasive compared to the collection of other tissues from live organisms and is possible under both laboratory and field conditions. Basic hematological parameters: hematocrit (Ht), hemoglobin concentration (Hb), erythrocyte count (RBC), leukocyte count (WBC), and blood smear can be obtained using a small amount of blood (about 200 µL is sufficient). Additional derived red blood parameters can also be calculated: mean corpuscular volume (MCV), mean corpuscular hemoglobin (MCH), and mean corpuscular hemoglobin concentration (MCHC) using Ht, Hb, and RBC values and appropriate formulas. Stained blood smears may be used for quantitative evaluation of erythrocyte and leukocyte populations to calculate the percentage of immature erythrocytes (erythroblasts), erythrocyte cellular and nuclear anomalies, differential leukocyte count, and thrombocyte count. These parameters are useful to evaluate erythropoietic activity, cytotoxic and genotoxic effects, and the status of the immune system. Hematological analysis is laborious, and to obtain reliable results requires skilled and experienced personnel since most measurements are performed using manual methods—all cells in fish blood are nucleated, and thus standard automatic analyzers applied in mammalian hematology cannot be used. This may be overcome by using modern veterinary analyzers that can be adjusted to work with fish blood [1,2,3].

Toxic agents, e.g., metal ions, pesticides, or other anthropogenic aquatic pollutants, as well as pharmaceuticals such as immunomodulators, antimicrobial and antiparasitic therapeutics, or anesthetics, were proven to cause hematological changes in fish [4,5,6,7,8,9,10]. To observe toxicity-induced alterations, it is necessary to provide reference values. Unfortunately, as in all poikilothermic vertebrates, the internal environments of fish are variable and considerably affected by external conditions, and it is very difficult to establish reliable hematological reference values for a species since their ranges are very wide [11,12,13]. Witeska et al. [14] summarized the data obtained over eight years from 146 clinically healthy juvenile individuals of *Cyprinus carpio* used as controls in various studies carried out under similar environmental conditions, and the results showed different levels of variability in various hematological parameters; some of them were stable (e.g., frequency of lymphocytes), most were moderately variable (e.g., hemoglobin concentration and red blood cell count), while others turned out to be highly variable (e.g., thrombocyte count). Therefore, some parameters have better biomarker potential than others. According to Ahmed et al. [15], variability of hematological parameters in fish results from the variable internal environment of the fish and the changes of environmental factors. It should be emphasized that reference (normal) values should be obtained in each particular experiment or field study as values for a control group of fish not exposed to the pollutants, kept in a tank with clean water, or sampled from a non-polluted site at the same time as the exposed individuals.

Hematological alterations in fish subjected to xenobiotics may be different and depend on the toxic agent, its concentration and time of exposure, environmental conditions, and intrinsic factors such as fish species, age, and size [1,15,16]. The observed hematological changes may indicate adaptive response of organisms to toxicity, show damage, or both; therefore, they are sometimes difficult to interpret. Hematological responses of fish to toxic exposures may also differ among the species and life stages that differ in their sensitivity to environmental impacts. Most toxic agents induce general and nonspecific stress and oxidative stress response, as well as cytotoxic effects and compensatory reactions.

### 1.1. Changes in Red Blood Cell Parameters Induced by Chemicals

Red blood cell parameters include hematocrit (Ht), hemoglobin concentration (Hb), erythrocyte count (RBC), mean corpuscular volume (MCV), mean corpuscular hemoglobin (MCH), and mean corpuscular hemoglobin concentration (MCHC). Additional information about the red blood cell system may be obtained by microscope analysis of blood smears: percentage of immature erythrocytes (erythroblasts) as an indicator of erythropoietic activity and percentage of various erythrocyte anomalies including cellular and nuclear deformities as indicator of cytotoxicity and genotoxicity. The observed toxicity-induced changes may be different and show an increase or decrease in the values of all or some red blood cell parameters, indicating the changes in oxygen transport capacity. An increase in Ht, Hb, RBC, or MCV may occur as a compensatory response, facilitating oxygen transport when a toxic agent causes a general stress, impairs gas exchange by affecting gill epithelium, or activates fish metabolism, e.g., increasing detoxification pathways. According to Carvalho and Fernandes [17], copper caused an increase in Ht, Hb, and RBC values in *Prochilodus scrofa*, and the authors concluded that these changes indicated ionoregulatory or respiratory disturbances that caused an increase in energetic metabolism to restore the impaired functions. Dias de Moraes et al. [18] reported an increase in Ht, Hb, and RBC values of *Brycon amazonicus* following acute exposure to pyrethroid insecticide cypermethrin and concluded that it was an adaptive response to hypoxia due to gill morphological changes that probably impaired the oxygen uptake and red blood cells were probably released from the spleen or hematopoietic tissue. Azithromycin induced a dose-dependent increase in Ht and Hb in *Oreochromis niloticus* [19]. An increase in Ht and MCV was observed by Rożyński et al. [20] in *Perca fluviatilis* after anesthesia with etomidate. Guimaraes et al. [21] reported a concentration-related increase in Ht, Hb, and RBC in *Oreochromis niloticus* fed diets supplemented with various levels of vitamin A.

A decrease is observed if a toxic agent causes damage to circulating erythrocytes; direct hemolysis in circulation or shortened erythrocyte life span, or/and impairs erythropoiesis. Such changes are described as an anemic response. However, usually it is difficult to identify an exact cause of anemia. Ligina et al. [22] reported a decrease in Ht, RBC, and Hb, and an MCV increase in *Anabas testudineus* treated with acrylamide. Yonar et al. [23] found a decrease in RBC, Hb, Ht, MCV, MCH, and MCHC in *Cyprinus carpio* subjected to organophosphate insecticide chlorpyrifos and explained anemia with possible impairment of erythropoietic activity, osmoregulatory disturbances, or accelerated eryptosis in the hematopoietic tissue. According to Jayaprakash and Shettu [24], pyrethroid insecticide deltamethrin caused a significant decrease in Ht, Hb, RBC, MCV, and MCHC of *Channa punctatus*. The authors explained the anemic response with impaired iron absorption and/or inhibition of enzymes involved in hemoglobin synthesis. Haider and Rauf [25] reported a significant decrease in RBC, Hb, HCT, MCV, and MCH compared to the control in *Cirrhinus mrigala* after chronic exposure to organophosphate insecticide diazinon. According to the authors, the anemic response might have been attributed to the failure or suppression of the hematopoietic system of the fish. Javed et al. [26] observed macrocytic hypochromic anemia (considerable decrease in Ht, Hb, RBC, and MCHC accompanied by increase in MCV and MCH), causing a strong impairment of oxygen carrying capacity in *Channa punctatus* subjected to industrial effluents containing metal ion mixture Co, Cr, Cu, Fe, Mn, Ni, and Zn, and interpreted anemia with the inhibition of erythropoiesis. Ko et al. [27] reported a concentration-related decrease in Ht, Hb, and RBC of *Platichthys stellatus* intoxicated with hexavalent chromium and attributed these changes to hemophilia, osmoregulatory disturbances, or a direct adverse effect of Cr on hematopoietic stem cells. According to Bishkoul et al. [28], anesthesia with MS-222 caused a decrease in Hb, Ht, and RBC in *Acipenser ruthenus*. According to Dawood et al. [29], deltamethrin caused a decrease in Hb and RBC in *Oreochromis niloticus*. A decrease in Ht, Hb, and RBC in the same fish species after oxytetracycline exposure was also reported by Omoregie and Oyebani [30].

Microscope analysis of the red blood cell population is another useful tool to evaluate the effects of toxic agents on fish. In Pappenheim stained smears, erythrocytes are well visible as regular elliptical cells with uniform acidophilic cytoplasm and centrally located elliptical basophilic nucleus. Thus, any changes in the cell or nucleus shape and staining properties may be considered anomalies. On the other hand, erythroblasts may be also observed, usually as smaller, rounder, and polychromatophilic (purplish) cells, with a larger and less condensed nucleus compared to the mature erythrocytes. A percentage of erythroblasts can be calculated as a good indicator of erythropoietic activity. Pala and Dey [31] reported an increase in the frequency of abnormal erythrocytes (crenated, ruptured, and contracted cells, echinocytes, spherocytes, lobopodial projections, and membrane internalization) in *Channa gachua* exposed to municipal wastewater. Kaur and Kaur [32] reported various nuclear and cellular abnormalities detected using light and scanning electron microscopy in *Labeo rohita* subjected to acute and subchronic exposure to Basic violet-1 dye. The authors observed erythrocyte anomalies before the appearance of other toxicity symptoms such as behavioral anomalies or mortality. A high frequency of micronuclei and lobed nuclei were observed in *Channa punctatus* exposed to thermal power plant effluent containing a mixture of heavy metals [26]. According to Farag and Alagawany [33], nucleated erythrocytes of fish can be used to evaluate genotoxicity of xenobiotics using various assays: comet, DNA fragmentation, or micronucleus test. Erythrocytes may be also useful for evaluation of toxicity-induced apoptosis, oxidative stress, and other cellular damage measures [34,35,36].

### 1.2. Changes in White Blood Cell Parameters Induced by Chemicals

Leukocyte count (WBC) and leukogram also called as differential leukocyte count (DLC—percentage of various types of leukocytes) are the most commonly used indicators of fish immune potential. Toxic agents often affect leukocyte count and, similar to the case of red blood parameters, an increase or decrease may be observed. An increase in WBC (leukocytosis) is usually interpreted as activation of the immune response due to tissue damage by a toxic agent and often neutrophilia or/and monocytosis is observed, indicating an inflammatory response. On the other hand, leukopenia (decrease in WBC) is attributed to a toxicity-induced general stress response (causing particularly lymphopenia and an increase of neutrophil to lymphocyte ratio) or specific toxic action affecting circulating leukocytes or leukopoiesis resulting in immunosuppression. Leukocytosis (increase in numbers of all types of leukocytes) was reported by Ligina et al. [22] in *Anabas testudineus* during intoxication with acrylamide. According to Zahran et al. [37], exposure to insecticide chlorpyrifos caused leukocytosis in *Oreochromis niloticus*. The numbers of both main leukocyte populations increased: neutrophils and lymphocytes but the increase in neutrophils was more pronounced (neutrophilia). The authors interpreted these changes as compensation action to the potential compromised immune functions. Bujjamma and Padmavathi [38] reported a concentration-dependent increase in WBC in *Heteropneustes fossilis* subjected to cadmium exposure. According to the authors, this might have resulted from immunomodulation caused by cadmium-induced tissue damage. According to Javed et al. [26], who observed increased WBC in *Channa punctatus* subjected to power plant effluent containing mixture of metal ions, leukocytosis was related to the magnitude of damage and stress induced by heavy metals which might have resulted in the stimulation of immunological defense. Leukocytosis caused by azithromycin was reported by Shiogiri et al. [19] in *Oreochromis niloticus*. According to Mahboub et al. [39], *Oreochromis niloticus* exposed to Hg showed leukocytosis, lymphopenia, and neutrophilia accompanied by impaired immune functions. Oluah et al. [40] observed leukocytosis, lymphocytosis, neutropenia, and monocytopenia in *Clarias gariepinus* exposed to the herbicide Ronstar.

Leukopenia was reported in *Sebastes schlegelii* exposed to ammonia [41] and, according to the authors, it was induced by stress. Tavares-Dias et al. [42] observed a decrease in WBC (both lymphocyte and neutrophil count) in *Colossoma macropomum* exposed to copper. *Oreochromis niloticus* exposed to nonylphenol showed leukopenia related to lymphopenia, and monocytopenia which indicates immunosuppression [43]. Lymphopenia and granulocytosis resulted from exposure of *Coregonus lavaretus* to anesthetic propofol [44] which indicates stress. Leukopenia was reported by Dawood et al. [29] in *Oreochromis niloticus* exposed to deltamethrin and after oxytetracycline treatment by Omoregie and Oyebani [30]. Maklakova et al. [45] observed monocytosis and neutropenia in *Oncorhynchus mykiss* subjected to benzylpenicillin or oxytetracycline treatments.

### 1.3. Changes in Thrombocyte Count Induced by Chemicals

Thrombocyte count (TC), being an estimator of blood coagulation and also a marker of nonspecific immune functions, is rarely measured. It is usually obtained indirectly by counting thrombocytes in a smear and then estimating TC from their proportion to leukocytes or erythrocytes and WBC or RBC, respectively. Thrombocyte count in fish is very variable [14] and they are sometimes included in leukocyte population as they are involved in both blood coagulation and defense mechanisms [46,47]. Thrombocyte count may show different alterations due to intoxication but due to high individual variability of this parameter, the results are often inconclusive.

According to Witeska and Kościuk [48], *Cyprinus carpio* subjected to acute exposure to Zn showed stress-related thrombocytosis. Lemly [49] observed thrombocytosis in *Lepomis cyanellus* from Belews Lake contaminated with selenium. Corredor-Santamaria et al. [50] reported thrombocytosis in *Astyanax bimaculatus* and *Aequidens metae* from Ocoa River polluted with domestic and industrial wastewater. The authors explain it with a pollution-related increase in the defense response. Fredianelli et al. [51] reported thrombocytopenia in *Rhamdia quelen* sublethally intoxicated with pesticide fipronil and explained it with stress-related cortisol secretion and its action reducing the quantity and quality of thrombocytes. Khan et al. [52] reported different reactions of thrombocytes to glyphosate and atrazine; the first herbicide induced an increase in TC, while the latter, a decline. Thrombocytopenia was also observed by Omoregie and Oyebani [30] in *Oreochromis niloticus* after oxytetracycline treatment.

Hematological analysis is one of the tools commonly used to evaluate the health and welfare of fish, both under aquaculture conditions and in scientific studies, to assess the influence of environmental factors on fish [1,16]. The study conducted by Bojarski et al. [53] demonstrated that hematological indices were the most sensitive and reliable biomarkers of exposure of *Cyprinus carpio* to the herbicide Roundup. Far fewer changes in comparison to hematological ones were observed in the blood biochemical parameters, while microstructure of the analyzed organs (gills, liver, trunk kidney) was unchanged. Thus, the authors concluded that hematological analysis is a basic and necessary tool in evaluation of the effects of Roundup exposure (in the case of *Cyprinus carpio*). Undoubtedly, hematological parameters are sensitive and early indicators of physiological alterations, and they provide valuable and extensive information about the effects of various chemicals in fish. Hematological indices inform us about oxygen transport capacity, immune status, stress response, cytotoxicity, and genotoxicity. Blood sampling is relatively noninvasive and easy even under field conditions. Evaluation of basic hematological parameters is inexpensive and does not require sophisticated laboratory equipment but to obtain reliable results must be performed and interpreted by highly skilled personnel. The observed changes are usually nonspecific and thus do not help to identify a cause of intoxication in case of exposure to unknown pollutants. To evaluate hematotoxicity of a particular compound or polluted environment, it is necessary to compare the results with the values obtained at the same time in the same fish species living under control conditions since unambiguous reference hematological values for the vast majority of fish do not exist. Nevertheless, taking into consideration the advantages of hematological analysis, we recommend this method for evaluation of toxicity in fish.

## 2. Hematopoietic Analysis in Fish Toxicology

As demonstrated, hematological analysis is widely applied in scientific research. In order to make a better use of the biological material and to obtain a broader knowledge of the mechanisms of the observed hematological changes, expanding standard hematological tests with other laboratory techniques deserves consideration in each scientific experiment involving fish. A good way of supplementing routinely determined hematological parameters is assessing the activity of hematopoietic tissue. To the best of our knowledge, very few studies concerning these issues have been published so far. It may be a result of the fact that the hematopoietic analysis is more complicated and laborious than determination of basic hematological parameters, cannot be performed in live fish, and is often associated with higher costs in comparison to standard hematological analysis (due to the fact that immunocytochemical staining used during the analysis is relatively expensive).

In most teleost fishes (Teleostei), the head kidney, pronephros, is the dominant hematopoietic organ and the reservoir of blood cells [54,55,56,57,58,59,60,61,62]. It consists of blood-forming, immune, and endocrine tissues that are involved in the production of blood cells, antibodies, cortisol, and catecholamines [63]. The available works show that the basic hematopoietic structures and mechanisms, and all hematopoietic precursor cells in fishes are very similar to those of other vertebrates [57,58,64]. Histology and ultrastructure of hematopoietic tissues of fish head kidney are quite well described [56,65,66,67,68,69,70,71,72].

In the Pappenheim stained hematopoietic tissue smears, the blood precursor cells may be easily identified based on their morphology: size, shape, staining properties, type of cytoplasmic granularity and nucleus size, shape, and position. Unfortunately, in morphological description, synonyms are used by various authors [54,57,58,71,73,74,75,76] which makes the comparison of the results difficult. After identification of the cells (about 20 types of cells usually can be found), they may be grouped into the main cell lineages (early blasts, erythroid, granuloid: neutrophilic, basophilic and eosinophilic, lymphoid, monocytoid, and thrombocytoid), each including various developmental stages [65,76,77,78,79,80]. Quantitative evaluation of hematopoietic tissue cellular composition includes calculation of percentages of each type of precursor and mature cell in the total number of blood cells examined. However, the data on the cellular composition of hematopoietic organs in teleosts are scarce and incomplete [58,62,74,75,79,80,81,82,83]. Comparison of data obtained by various authors using the same criteria of cell identification show that cellular composition of head kidney hematopoietic tissue of various fish species is similar. However, quantitative differences were observed [80]. The composition of fish hematopoietic tissue is very variable (more than the peripheral blood) and depends on various intrinsic and environmental factors such as fish age, sex reproductive cycle, nutrition, pathogens, water temperature and other physicochemical water parameters, aquatic pollution, and other stressors. It is obvious that the composition of hematopoietic tissue and the frequency of various cell lineages also vary even in the same fish species and reflect the adaptation of organisms to variable environmental conditions. This is an advantage that makes interspecific comparisons and use of the hematopoietic system as an indicator of environmental impacts possible. On the other hand, the differences may occur even within the same species if different methodologies and cell nomenclature are used. Such discrepancies indicate the need for the unification of nomenclature and the cell identification criteria in the study of hematopoietic organs [79].

There is no doubt that hematopoietic tissue of the head kidney is very active. Hematopoietic activity of head kidney tissue is related to the rate of proliferation of pluripotent hematopoietic stem cells and early precursors of all blood cell lines, their differentiation and maturation, as well as to the rate of hematopoietic cell apoptosis. These processes are key factors that determine the efficiency of hematopoiesis. Hematopoietic stem cell renewal and precursor cell proliferation are counterbalanced by apoptosis in functionally inactive or terminally differentiated cells [84]. Proliferating cells show the expression of proliferating cell nuclear antigen (PCNA)—a protein present only in cells undergoing mitosis. The marker protein of apoptotic cells is caspase 3—an enzyme participating in degradation of nuclear and cytoplasmic proteins during this process. Caspase 3 is commonly defined as effector caspase [85]. Proliferative and apoptotic activity in the head kidney can be evaluated using immunocytochemical staining to visualize PCNA-positive cells (indicating proliferative activity) and caspase 3-positive cells (indicating apoptotic activity). Evaluations of both precursor cell proliferation and apoptosis rate are applied in hematological studies to evaluate the rate of cell turnover and hematopoietic activity [86,87]. Hematopoietic activity can be calculated as the ratio of proliferating to apoptotic cells. Both fish PCNA and caspase 3 have been proven to react with mammalian (mouse or rabbit) monoclonal antibodies, and these antibodies were successfully used for evaluation of proliferation and apoptosis of various cells in *Oreochromis niloticus* [88], *Thalassoma pavo* [89], and *Salmo salar* [90].

The collected data prove that toxic substances enhance the rate of hematopoietic precursor cell’s apoptotic destruction. On the other hand, the hematopoietic system of fish shows high homeostatic potential and tends to compensate for cell loss by the activation of mitotic divisions. Som et al. [81] reported an increase in apoptotic rate of hematopoietic precursors in copper-exposed *Labeo rohita*, while the proliferation rate was elevated under sublethal conditions and reduced in fish subjected to lethal exposure. Cadmium significantly reduced overall hematopoietic potential of head kidney tissue; the proliferation rate did not change but the rate of apoptosis significantly increased in the cadmium-exposed fish [91]. Berntssen et al. [92] and Garcia-Santos et al. [93] reported an increase in cell proliferation rate in cadmium-exposed *Salmo salar* and *Sparus aurata*, respectively, and according to these authors, it might have been a protective mechanism reducing the adverse effects of metal on fish tissues. Kondera et al. [94] observed a significant increase in the rate of cell proliferation in *Cyprinus carpio* exposed to Roundup at concentrations 0.5 and 5.0 mg/L.

There is very little scientific data available on the toxic effects of environmental factors (natural and anthropogenic) on the cellular structure of fish head kidney hematopoietic tissue [95,96,97,98]. It can, therefore, be assumed that due to the high rate of cell exchange in the hematopoietic tissue of the fish head kidney, its sensitivity to various factors that disturb homeostasis is high [99]. Studies of hematopoietic tissue are seldom included in the evaluation of the physiological effects of toxic agents in fish, although this knowledge can be helpful in interpreting the changes in peripheral blood and the immune system of fish. Supplementing the standard hematological analysis with analysis of the cellular composition of hematopoietic tissue allows to obtain more complete information about the changes in fish organisms than peripheral blood analysis alone. Changes in the values of peripheral blood parameters are often non-linear, which may indicate the effect of particular factors on various physiological processes. Analyses of hematopoietic tissue can help clarify whether hematological changes observed in fish result from the direct action of environmental factors (e.g., toxic substances present in aquatic environment) on circulating blood cells or from disorders in hematopoietic processes (indirect action).

Kondera and Witeska [91] noted that the high frequency of abnormal erythrocytes observed in peripheral blood of *Cyprinus carpio* after long-term exposure to 0.65 mg/L of Cd was accompanied by increased erythropoiesis, while the minor effect of cadmium observed after short-term (6.5 mg/L) exposure did not activate erythropoiesis. All cadmium-exposed fish showed a decrease in lymphocyte frequency in head kidney tissue and a considerable reduction in blood phagocyte metabolic activity. The authors interpreted these changes as compensation for erythrocyte loss due to metal-induced cytotoxicity by activation of erythropoiesis. Reduced hematopoietic potential and a shift towards erythropoiesis to compensate for erythrocyte loss in fish subjected to long-term cadmium exposure probably caused a reduced supply of phagocytes (neutrophils and monocytes) to the blood. However, a reduction of phagocyte activity also occurred in fish after a short-term Cd exposure, in which an increase in WBC took place and no changes in blood phagocyte levels were observed. This indicates that cadmium affects not only the number but also the function of phagocytes. A reduction of the Hb concentration in *Cyprinus carpio* peripheral blood after exposure to 0.5 and 5.0 mg/L of Roundup may indicate an anemic response was not accompanied by a decrease in RBC or increased erythrocyte damage or hemolysis. On the other hand, higher hematopoietic activity accompanied by a higher frequency of head kidney erythroblasts in Roundup-exposed fish compared to control individuals may indicate accelerated erythropoiesis, probably in response to a shortening of erythrocyte life span [94]. The cellular composition and activity of fish hematopoietic tissue can be used as biomarkers and they may provide early warning signs of environmental contamination [91,94]. Moreover, the study by Kondera et al. [82] demonstrated that hematopoietic parameters were even more responsive to antibiotic treatment than peripheral blood indices. It was also documented that exposure to xenobiotics may lead to changes in ultrastructure of *Cyprinus carpio* hematopoietic tissues [100,101].

## 3. Conclusions

Hematological variables are sensitive and reliable indicators of environmental impacts on fish, including toxic agents. They may show either destructive effects of toxicity such as anemia and immunosuppression, or compensatory effects such as an increase in blood oxygen transport capacity or inflammation. Often, hematological changes in fish subjected to toxicity reflect a general, nonspecific stress response and are difficult to conclude about their mechanisms. The cellular composition and activity of hematopoietic tissue may provide additional information about physiological effects of toxicity and a complex hematological and hematopoietic analysis may show a more complete picture. Therefore, changes in the cellular composition and activity of hematopoietic tissue can be used as an important additional biomarker, complementary to the routine basic hematological tests. However, due to the high variability of the fish hematological and hematopoietic parameters and the lack of unambiguous reference values, it is important to provide an appropriate control group in each experimental or field study.

## Data Availability

Dataset supporting the reported results can be found at Web of Science or PubMed.
